# Toward an embodied science of intersubjectivity: widening the scope of social understanding research

**DOI:** 10.3389/fpsyg.2015.00234

**Published:** 2015-03-02

**Authors:** Ezequiel A. Di Paolo, Hanne De Jaegher

**Affiliations:** Logic and Philosophy of Science, IAS-Research Centre, University of the Basque CountryDonostia/San Sebastián, Spain

**Keywords:** intersubjectivity, social interaction, embodiment, enaction, methodology

The study of human social phenomena in their proper scope demands the integrated effort of many disciplinary traditions. This fact is widely acknowledged but rarely acted upon. It is in practice often difficult to cross disciplinary boundaries, to communicate across different vocabularies, research goals, theories and methods. The aim of this Research Topic has been to make some progress in stepping across these borders.

Not attempting this crossing in a subject as multi-faceted as intersubjectivity inevitably binds us to remain within self-enclosed conceptions. By this we mean a bundle of self-reinforcing perspectives, hypotheses, experimental methods, debates, communities and institutions. Traditional ways of thinking about social cognition frame the questions that are deemed worth researching. These all revolve around the issue of how we figure out other minds, assuming that other people's intentional states are hidden, private and internal. The proposed answers rely only on how the perceived indirect manifestations of other people's mental states are processed by individual cognitive mechanisms (Van Overwalle, 2009).

We would like to raise, instead, the question of what an embodied science of intersubjectivity would look like if we were to start from different premises than those that delimit classical approaches to social cognition. For doing this, we thought the time was ripe for bringing together work that crosses disciplinary boundaries and informs us about different conceptions of how people understand each other and act and make meaning together.

The move is timely. The internalist assumptions in social cognition research are beginning to shift. We have more and better tools to explore the role of interactive phenomena and interpersonal histories in conjunction with individual processes (Dumas et al., 2010; Di Paolo and De Jaegher, 2012; Konvalinka and Roepstorff, 2012; Schilbach et al., 2013). This interactive expansion of the conceptual and methodological toolkit for investigating social cognition, we now propose, can be followed by an expansion into wider and deeply-related research questions, beyond (but including) that of social cognition narrowly construed.

Our social lives are populated by different kinds of cognitive and affective phenomena apart from figuring out other minds. They include acting and perceiving together, verbal and non-verbal engagement, experiences of (dis-)connection, relations in a group, joint meaning-making, intimacy, trust, secrecy, conflict, negotiation, asymmetric relations, material mediation of social interaction, collective action, contextual engagement with socio-cultural norms, etc. These phenomena are often characterized by a strong participation by the cognitive agent, in contrast with the spectatorial stance of social cognition (Reddy and Morris, 2004; De Jaegher and Di Paolo, 2007). We use the broader notion of *embodied intersubjectivity* to refer to this wider set of questions.

Forty-two contributions to this Research Topic explore several of these themes. They combine ideas and methods from psychology, neuroscience, philosophy of mind, phenomenology, psychiatry and psychotherapy, social science, and language studies. The number of contributions confirms our suspicions that there is a genuine interest in embodied intersubjectivity.

All of the contributions in some way or other move beyond traditional cognitivist perspectives. Here we can simply highlight some of the most interesting ways in which this happens. As already mentioned, there is a recent trend to investigate the dynamics of actual interactive encounters between people. Several empirical studies in this Research Topic continue further along this line. They look at interactive encounters using methods such as thermal imaging, interactive virtual environments, or 1/f noise analysis, or combine existing methods with novel theoretical starting points.

Other work looks at aspects of embodied social understanding which are pertinent even in the absence of ongoing interaction. These include the richness of body kinematics, affect regulation, and life-story analysis. A few contributions focus on how embodied and interactive perspectives impact on developmental research. They study real-life interactions between infants and their care-givers in various contexts (infant pick-up, book sharing, pointing, cooperation, and expressiveness during play in chimpanzees). Aspects of psychopathology are explored also from an embodied intersubjective angle, inspiring research on intra- and inter-personal emotion regulation, social affordances, personal biography, and therapeutic play, and their effects on somatic symptom disorders, autism, and schizophrenia.

Broadening the scope of relevant questions for embodied intersubjectivity inevitably means including research on language. Many of the contributions make headway on this matter, questioning the notion of the common ground, the role of conformity in social understanding, the processes involved in the activity of reading texts, and the links between conversational coordination and meaning-making. Others investigate the participatory nature of understanding narratives, and the role of organizational, temporal, and inter-affective aspects in language. Similar advances can be made in the area of connecting the cognitive and the social sciences. This is a very fruitful but still largely unexplored territory. A discussion is offered along Marxist lines concerning the interaction between categories of understanding and modes of social exchange and production. And the lessons of embodied/enactive approaches to intersubjectivity are summoned to contribute to understanding the phenomenological and social effects of solitary confinement.

Finally, some contributions elaborate theoretical and methodological implications and concepts, and in this way contribute to shaping the core of an embodied science of intersubjectivity. Methodological issues include whether dynamical systems concepts can bridge the multiple scales involved in social understanding, from the biological and neural to the personal, interactive and societal, how second person perspectives in cognitive science can help psychopathology research, and whether techniques used in theater can refine intuitions and theoretical concepts about interactive experience. Theoretical advances include radically embodied accounts of intersubjectivity that bring together conceptions from enactivism and ecological psychology, the notion of intersubjective time, and a socially embodied notion of the human self. Other discussions offer links between interpersonal interaction and phenomenal experience, between social normativity and conceptual abilities, or unearth the importance of opacity, i.e., the secret, silent or hidden aspects of personal experience, for understanding each other.

It is noteworthy, and especially satisfying, that many novel themes and questions emerged, several of them in some way related to personal meaning. To name a few: joy, secrecy, solitude, influence of capitalist mode of production on cognition, book sharing in infancy, the search for comprehensiveness and integrity in interacting, literature, and enactivism, ethics of care, shame in relation to interaction, and the interactive building blocks of culture and institutions.

Once again, we notice that the contributions to this Research Topic demonstrate the richness of enquiry and research work that is opened by the combination of novel methods and the bringing together of fields that traditionally work in isolation from each other. It also shows that criticisms of classical approaches as being sometimes too narrow are not just idle but point to genuinely new perspectives on concrete and everyday intersubjectivity that are opened to investigation.

## Conflict of interest statement

The authors declare that the research was conducted in the absence of any commercial or financial relationships that could be construed as a potential conflict of interest.
